# Increased age, bilirubin, international normalized ratio, and creatinine score to triglyceride ratio are associated with alcohol-associated primary liver carcinoma: a single-centered retrospective study

**DOI:** 10.1186/s12944-023-01888-y

**Published:** 2023-08-04

**Authors:** Xiaoqing Jia, Rong Li, Xiaoting Zhang, Tao Zhou, Dalong Sun, Na Yang, Zheng Luo

**Affiliations:** 1 Department of Gastroenterology, Qilu Hospital, Shandong University, 107 West Wenhua Road, Jinan, Shandong 250012, 250010 P.R. China; 2 Department of Geriatric Medicine, Qilu Hospital, Shandong University, 107 West Wenhua Road, Jinan, Shandong 250012, 250010 P.R. China

**Keywords:** Alcohol-associated liver diseases, Primary liver carcinoma, Epidemiology, Dyslipidemia, Triglyceride

## Abstract

**Background:**

This study analyzed the clinical features and biomarkers of alcohol-associated liver disease (ALD) to investigate the diagnostic value of age, bilirubin, international normalized ratio (INR), and creatinine (ABIC) score to triglyceride (TG) ratio (ABIC/TG) in ALD-associated primary liver carcinoma (PLC).

**Materials and methods:**

Data were collected from 410 participants with ALD, and the epidemiological and clinical records of 266 participants were analyzed. Participants were divided into ALD-without-PLC and ALD-associated-PLC groups. Relationships between clinical characteristics, biomarkers and ALD-associated PLC were estimated. Serum lipid levels and liver function were compared between ALD patients without PLC and patients with ALD-associated PLC. Scoring systems were calculated to investigate ALD severity. The robustness of the relationship was analyzed by the receiver operating characteristic (ROC) curve.

**Results:**

Age and dyslipidemia were more strongly associated with ALD-associated PLC than with ALD-without PLC, with AORs of 2.39 and 0.25, respectively, with *P* less than 0.05. Drinking time and average daily intake, ABIC score, and ABIC/TG ratio were significantly higher in the ALD-associated-PLC group than in the ALD-without-PLC group. The AUC for the ABIC/TG ratio predicting the incidence of PLC was 0.80 (*P* < 0.01), which was higher than that of the ABIC and TG scores alone; additionally, the specificity and Youden index for the ABIC/TG ratio were also higher, and the cutoff value was 6.99.

**Conclusions:**

In ALD patients, age, drinking time, and average daily intake were risk factors for PLC. Drinking time, average daily intake, TG and ABIC score have diagnostic value for ALD-associated PLC. The ABIC/TG ratio had a higher AUC value and Youden index than the ABIC score and TG level.

**Supplementary Information:**

The online version contains supplementary material available at 10.1186/s12944-023-01888-y.

## Background

Alcohol-associated liver diseases (ALDs) are induced by alcohol abuse and include liver hepatitis, fibrosis, cirrhosis and hepatocellular carcinoma (HCC) [1]. HCC is the most common type of primary liver carcinoma (PLC), accounting for 90% of PLC cases [2, 3]. Alcohol is a carcinogen because it contributes to 15–30% of HCC cases [4–6]. However, methods for surveilling patients with ALD to facilitate the early-stage diagnosis of PLC have yet to be established.

Alcohol impacts hepatic lipid flux, thus leading to the accumulation of lipids in hepatocytes [7]. Significantly lower values for serum lipids were observed in HBV- and HCV-induced liver fibrosis [8]. Research has shown that increased serum cholesterol accumulated in NK cells could activate their effector functions against hepatoma cells to inhibit malignant tumor growth in mice [9]. However, little research has explored the association between serum lipids and the etiology of ALD-associated PLC.

Alcoholic hepatitis severity was measured by several pretreatment scoring systems, including the Maddrey Discriminant Function (DF) scoring system, Model for End-stage Liver Disease (MELD) scoring system, age, bilirubin, international normalized ratio (INR), creatinine (ABIC) scoring system and Glasgow Alcoholic Hepatitis Score (GAHS) system [10–12]. The current study used these scores to evaluate the severity of ALD.

This study was performed by analyzing liver function, serum lipids and the severity of ALD to identify indicators or predictors of ALD-associated PLC in China.

## Methods

Information on participants from Jan. 1, 2017, to Dec. 31, 2021, was collected. The Independent Ethics Committees of Qilu Hospital approved the research protocol (No. KYLL-202202-005-1). All the information was collected from electronic medical records in the hospital. The hospital ethics committee approved the waiver statement of informed consent since the data were anonymous.

### Study population

Data were collected from Qilu Hospital medical records with diagnoses associated with ALD (including alcoholic hepatitis, alcoholic cirrhosis and hepatic carcinoma accompanied by alcoholic hepatitis) between January 2017 and December 2021. The exclusion criteria include:

1. Participants without complete clinical information;
2. Participants with other liver disease histories, including autoimmune hepatitis, primary sclerosing cholangitis, virus hepatitis and liver metastases cancer;
3. less than 18 years of age; and.
4. pregnant.

### Diagnoses of ALD

ALD was diagnosed according to the following criteria: (1) participants with a recent onset of jaundice of less than 3 months; (2) a long-standing history of alcoholism; and (3) moderately increased transaminase and increased bilirubin levels, which is consistent with the biological profile of alcoholic hepatitis [13].

The ALD-associated PLC group was collected from ALD participants who were diagnosed with PLC according to liver biopsy or imaging studies, including magnetic resonance imaging (MRI) and computed tomography (CT).

### Diagnosis of dyslipidemia

Dyslipidemia was diagnosed according to the following criteria: participants with higher TG levels (> 1.7 mmol/L), higher Cho levels (> 6.00 mmol/L), higher LDL-C levels (> 3.37 mmol/L), or lower HDL-C levels (< 0.8 mmol/L).

### Severity of ALD

Biochemical results, including total bilirubin (TBIL), direct bilirubin (DBIL), alkaline phosphatase (AKP), gamma-glutamyl transferase (GGT), aspartate transaminase (AST), alanine aminotransferase (ALT), prothrombin time (PT) and international normalized ratio (INR), were collected via routine laboratory tests upon hospital admission. The DF, MELD, ABIC and GAHS scores were all calculated to investigate the severity of ALD. The formulas for calculating each score are shown in Table 1 [10, 12, 14, 15].

**Table 1:** ALD scoring system formula

Scoring system	Formula
DF	4.69 × (PT - control PT) + bilirubin (mg/dL)
MELD	9.57 × log_e_ (creatinine, mg/dL) + 3.78 × log_e_ (bilirubin mg/dL) + 11.20 × log_e_ (INR) + 6.43
ABIC	(age × 0.1) + (bilirubin mg/dL × 0.08) + (INR × 0.8) + (creatinine mg/dL × 0.3)
GAHS	age (years) (< 50 = 1; ≥50 = 2) + leucocytes (10^9^/L) (< 15 = 1; ≥15 = 2) + urea (mmol/L) (< 5 = 1; ≥5 = 2) + PT ratio (< 1.5 = 1; 1.5–2.0 = 2; >2.0 = 3) + bilirubin (mmol/L) (< 125 = 1; 125–50 = 2; <250 = 3)

DF: Maddrey discriminant function; MELD: Model for End-stage Liver Disease; ABIC: age-bilirubin-international normalized ratio (INR)-creatinine; GAHS: Glasgow alcoholic hepatitis scores

### Classification of ALD

Participants were separated into the ALD-without-PLC group and the ALD-associated-PLC group.

### Demographic and clinical features

Demographic features were analysed, including age, sex, smoking, drinking time, daily alcohol intake, hypertension, diabetes mellitus (DM), cardiovascular disease (CVD), hypersensitivity, malignancy, and family history of chronic diseases.

Biochemical results of liver function were recorded to determine the severity of ALD. TG, Cho, HDL-C, and LDL-C were recorded to evaluate dyslipidemia in ALD. White blood cell (WBC) counts and creatinine were collected to calculate scoring systems.

### Statistical analyses

Continuous data are presented as the mean ± 1 standard deviation (mean ± SD) (normal distribution). Logistic regression models were performed in univariate analysis, and significant variables with *P* less than 0.1 were calculated in multivariable analysis. Spearman’s correlation was used to investigate the relationship between variables. The effective values of variables or models to predict the incidence of PLC were evaluated by using receiver operating characteristic (ROC) curves; subsequently, the area under the ROC curves (AUCs) and cutoff values were calculated. Statistical analyses were performed by using the SPSS software package v. 17.0 (SPSS, Chicago, IL). The results were shown graphically using GraphPad Prism 8.0. A *P* value below 0.05 indicates statistical nonsimilarity.

## Results

### Baseline characteristics

**Fig. 1:**
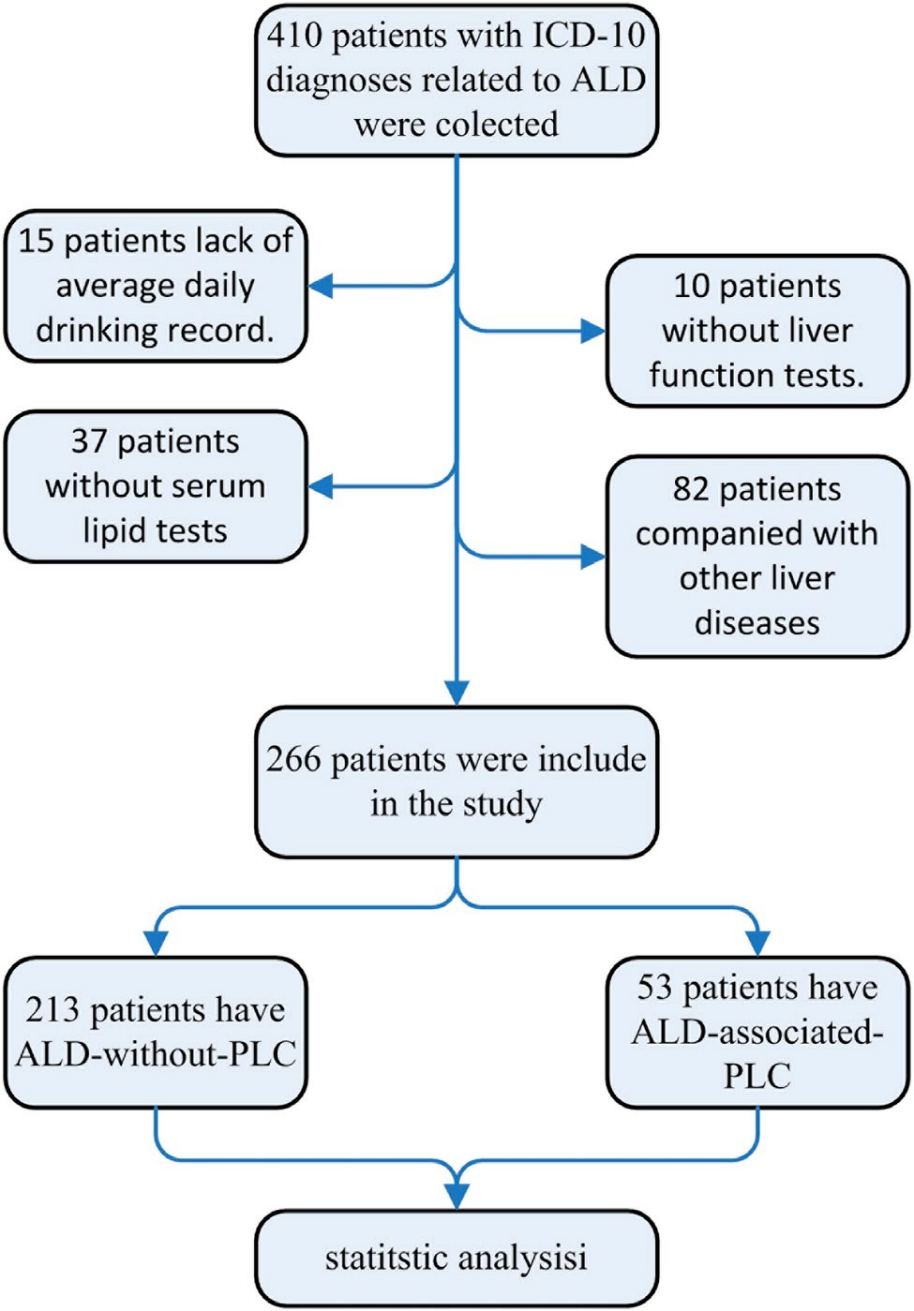
Flow chart of the study ALD: alcohol-associated liver disease; PLC: primary liver carcinoma

A total of 410 ALD participants were assessed, and 144 participants were excluded for various reasons. Information on 266 ALD participants was recorded and analyzed in the study. A total of 213 participants had ALD without PLC, and 53 participants had ALD-associated PLC (Fig. 1). Among ALD–without-PLC participants, 1.41% were female participants, and 98.59% were male participants, with an average age of 54.08. In the ALD-associated PLC group, 17 (32.08%) participants were diagnosed by biopsy of hepatocellular carcinoma, 20 (37.74%) participants were diagnosed by CT, and 16 (30.19%) participants were diagnosed by MRI of PLC. In the ALD-associated PLC group, 100.00% were males, and the average age was 62.4 (Table 2).

**Table 2:** Univariate and multivariate analyses of patient characteristics in the ALD-without-PLC group and ALD-associated-PLC group

Variable		ALD-without-PLC	ALD-associated-PLC	*P1*	OR(95%CI)	*P2*
Sex				1.00	-	-
	female (N%)	1.41	0.00			
	male (N%)	98.59	100.00			
Age		54.08 ± 10.82	62.4 ± 7.52	< 0.01	2.39(1.38–4.13)	0.01
	<60	65.26	37.74			
	≥ 60	34.74	62.26			
Smoking				0.53	-	-
	no (N%)	20.66	20.75			
	yes (N%)	79.34	79.25			
Hypertension				0.40	-	-
	no (N%)	72.77	83.02			
	yes (N%)	27.23	16.98			
Heart disease				0.21	-	-
	no (N%)	92.96	92.45			
	yes (N%)	7.04	7.55			
Diabetes				0.14	-	-
	no (N%)	87.79	71.70			
	yes (N%)	12.21	28.30			
Allergic history				0.98	-	-
	no (N%)	83.57	79.25			
	yes (N%)	16.43	20.75			
Familly chronic disease				0.51	-	-
	no (N%)	94.84	92.45			
	yes (N%)	5.16	7.55			
Dyslipidemia				< 0.01	0.25(0.14–0.44)	< 0.01
	no (N%)	59.15	75.47			
	yes (N%)	40.85	24.53			
PLC diagnose	biopsy		32.08			
	CT		37.74			
	MRI		30.19			

ALD: alcohol-associated liver disease; PLC: primary liver carcinoma; AOR: adjusted odds ratio. *P1*: *P* value for univariate analysis; *P2*: *P* value for multivariate analysis. AOR is adjusted for age and dyslipidemia

### Clinical and demographic characteristics

Participants with dyslipidemia had a lower risk of PLC than those without dyslipidemia (AOR = 0.25; 95% CI, 0.14–0.44; *P* < 0.01). Participants over 60 had an increased risk of PLC compared with those younger than 60 years old (AOR = 2.39, 95% CI, 1.38–4.13; *P* < 0.05). This study did not observe significant differences in smoking, CVD, hypertension, hypersensitivity, DM or family chronic diseases between the two groups (Table 2).

### Factors associated with ALD-associated PLC in the whole series

**Fig. 2:**
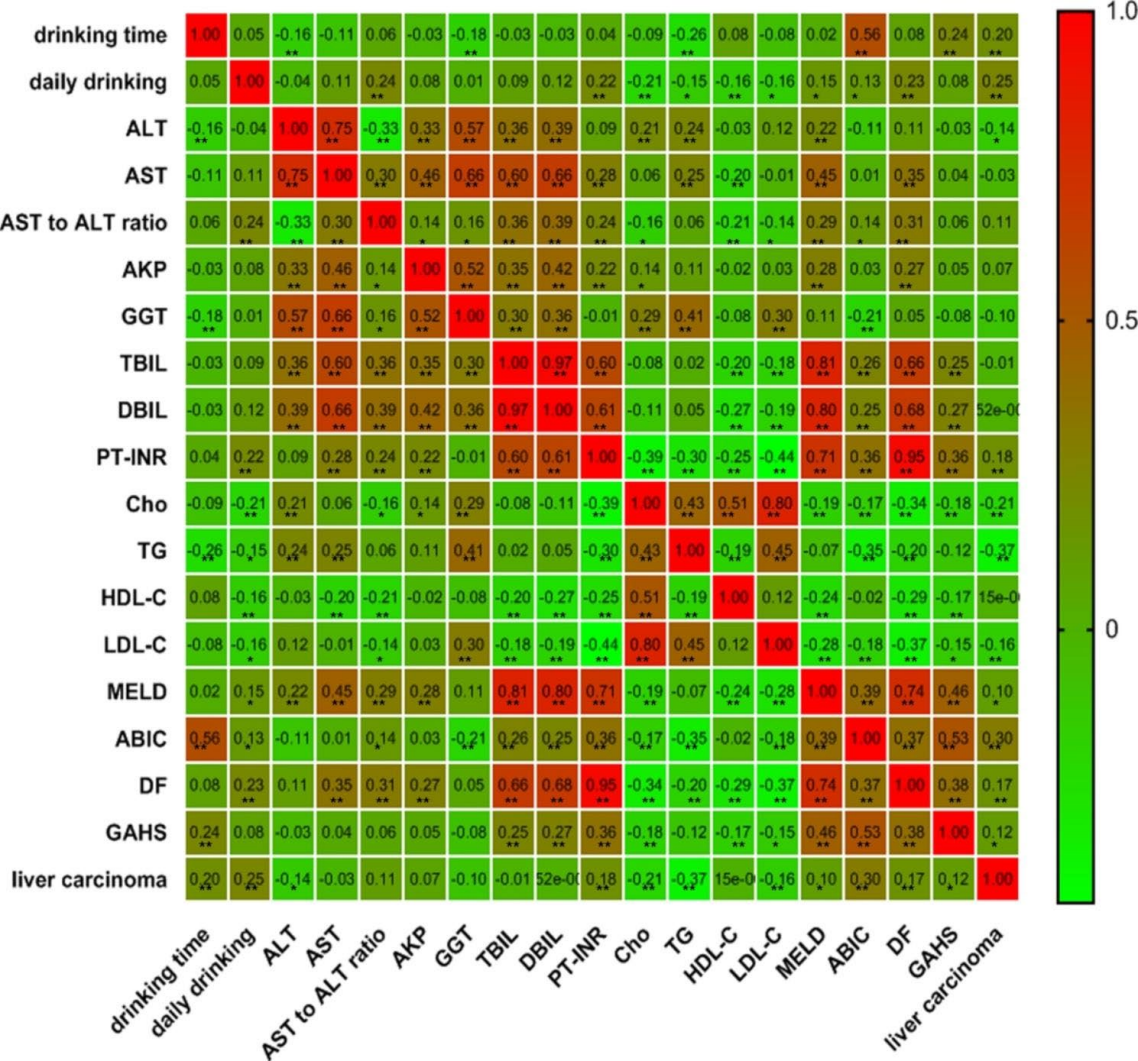
Correlation coefficients between factors and ALD-associated PLC. TG: triglyceride; Cho: cholesterol; HDL-C: high-density lipoprotein cholesterol; LDL-C: low-density lipoprotein cholesterol; ALT: alanine aminotransferase; AST: aspartate transaminase; GGT: gamma-glutamyl transferase; AKP: alkaline phosphatase; TBIL: total bilirubin; DBIL: direct bilirubin; PT-INR: international normalized ratio of prothrombin time; DF: Maddrey discriminant function; MELD: Model for End-stage Liver Disease; ABIC: age-bilirubin-international normalized ratio (INR)-creatinine; GAHS: Glasgow alcoholic hepatitis scores. * *P* ≤ 0.05; ** *P* ≤ 0.01

**Fig. 3:**
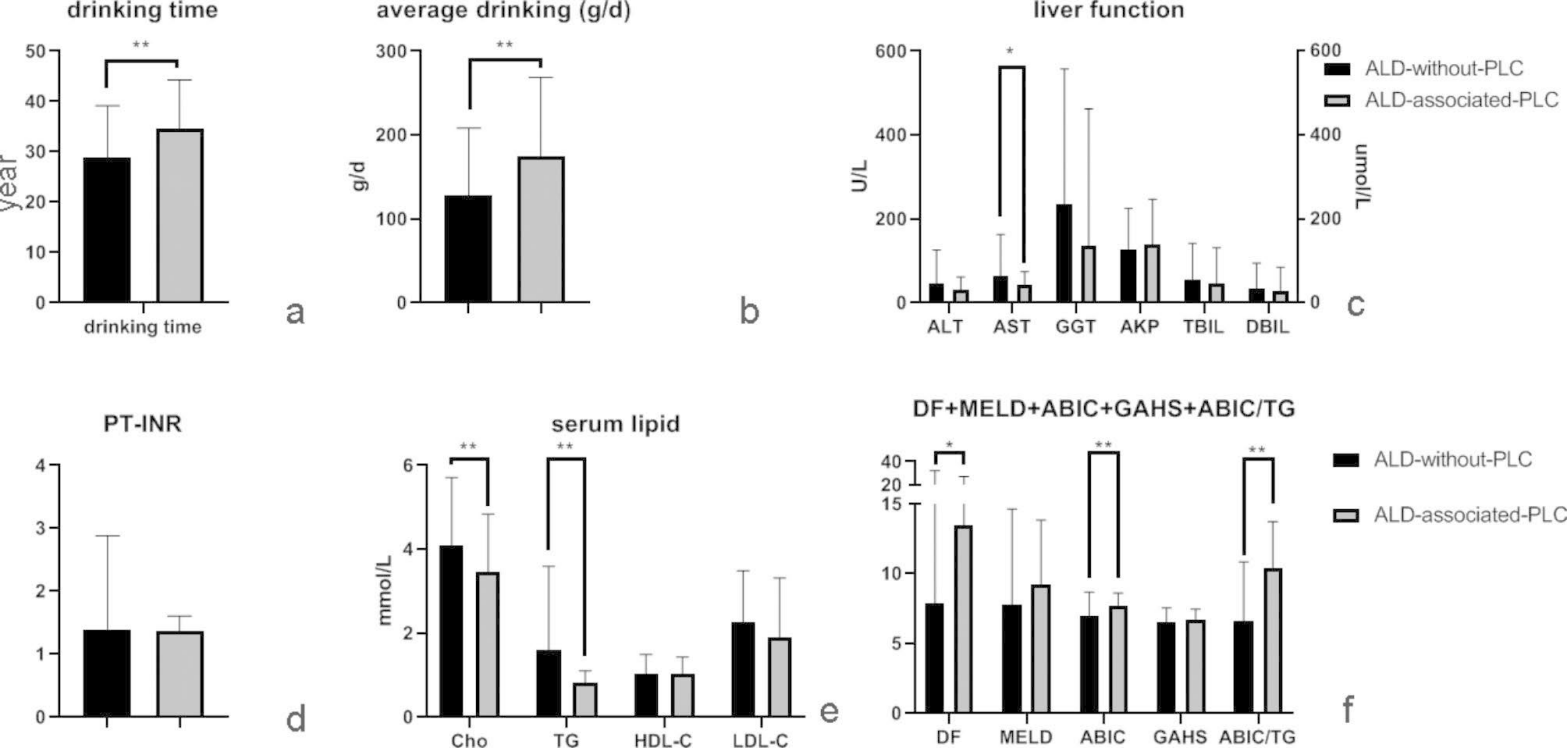
Factors in the ALD-without-PLC group and ALD-associated-PLC group. ALD: alcohol-associated liver disease; PLC: primary liver carcinoma; TG: triglyceride; Cho: cholesterol; HDL-C: high-density lipoprotein cholesterol; LDL-C: low-density lipoprotein cholesterol; ALT: alanine aminotransferase; AST: aspartate transaminase; GGT: gamma-glutamyl transferase; AKP: alkaline phosphatase; TBIL: total bilirubin; DBIL: direct bilirubin; PT-INR: international normalized ratio of prothrombin time; DF: Maddrey discriminant function; MELD: Model for End-stage Liver Disease; ABIC: age-bilirubin-international normalized ratio (INR)-creatinine; GAHS: Glasgow alcoholic hepatitis scoresass. (**a**) Drinking time in the ALD-without-PLC group and ALD-associated-PLC group. (**b**) Average daily drinking in the ALD-without-PLC group and ALD-associated-PLC group. (**c**) ALT, AST, GGT, AKP, TBIL and DBIL levels in the ALD-without-PLC group and ALD-associated–PLC group. (**d**) PT-INR level in the ALD-without-PLC group and ALD-associated-PLC group. (**e**) Cho, TG, HDL-C and LDL-C levels in the ALD-without-PLC group and ALD-associated-PLC group. (**f**) DF score, MELD score, ABIC score, GAHS score and ABIC/TG ratio in the ALD-without-PLC group and ALD-associated-PLC group. * *P* ≤ 0.05; ** *P* ≤ 0.01

Liver function, serum lipid levels and factors related to ALD, including drinking time and average daily intake, were analysed. The results showed significant positive correlations between the ABIC score and PLC and a negative correlation between TG and PLC incidence (correlation coefficient = 0.30, -0.37, *P* < 0.01). Drinking time and average daily intake were positively related to ALD-associated PLC (correlation coefficient = 0.20, 0.25, *P* < 0.01). All the data are shown in Fig. 2. Drinking time, average daily intake, serum lipid levels and liver function were further screened. Participants in the ALD-associated-PLC group had a significantly longer drinking time and increased average daily intake than participants in the ALD-without-PLC group. The AST, Cho and TG levels were lower in ALD participants with PLC than in ALD participants without PLC. The ABIC score of ALD-associated-PLC participants was significantly increased compared with that of ALD-without-PLC patients. All the data are shown in Table 3; Fig. 3.

**Table 3:** Clinical characteristics and biomarkers of patients in the ALD-without-PLC group and ALD-associated-PLC group

variables	ALD-without-PLC	ALD-associated-PLC	*P*
drinking time (years)	28.91 ± 10.22	34.57 ± 9.7	< 0.01
average daily drinking (g/d)	127.85 ± 80.35	175.47 ± 93.07	< 0.01
ALT (U/L)	47.14 ± 79.31	30.98 ± 30.99	0.15
AST (U/L)	63.96 ± 99.14	44.58 ± 29.47	0.01
GGT (U/L)	234.98 ± 322.19	136.26 ± 326.18	0.05
AKP (U/L)	128.67 ± 96.71	139.45 ± 107.05	0.48
TBIL (umol/L)	56.93 ± 84.79	47.68 ± 83.98	0.48
DBIL (umol/L)	34 ± 60.96	27.08 ± 58.14	0.46
PT-INR	1.4 ± 1.48	1.37 ± 0.24	0.88
AST to ALT ratio	1.74 ± 0.98	1.9 ± 0.71	0.41
Cho (mmol/L)	4.1 ± 1.61	3.46 ± 1.38	< 0.01
TG (mmol/L)	1.61 ± 1.99	0.82 ± 0.29	< 0.01
HDL-C (mmol/L)	1.03 ± 0.46	1.02 ± 0.42	0.92
LDL-C (mmol/L)	2.27 ± 1.22	1.9 ± 1.42	0.06
DF	7.86 ± 24.49	13.49 ± 13.64	0.03
MELD	7.81 ± 6.83	9.2 ± 4.63	0.08
ABIC	6.96 ± 1.71	7.74 ± 0.88	< 0.01
GAHS	6.54 ± 1.01	6.75 ± 0.7	0.07
ABIC score/TG (L/mmol)	6.59 ± 4.25	10.44 ± 3.27	< 0.01

ALD: alcohol-associated liver disease; PLC: primary liver carcinoma; TG: triglyceride; Cho: cholesterol; HDL-C: high-density lipoprotein cholesterol; LDL-C: low-density lipoprotein cholesterol; ALT: alanine aminotransferase; AST: aspartate transaminase; GGT: gamma-glutamyl transferase; AKP: alkaline phosphatase; TBIL: total bilirubin; DBIL: direct bilirubin; PT-INR: international normalized ratio of prothrombin time; DF: Maddrey discriminant function; MELD: Model for End-stage Liver Disease; ABIC: age-bilirubin-international normalized ratio (INR)-creatinine; GAHS: Glasgow alcoholic hepatitis scores

### Prognostic value of the factors

**Fig. 4:**
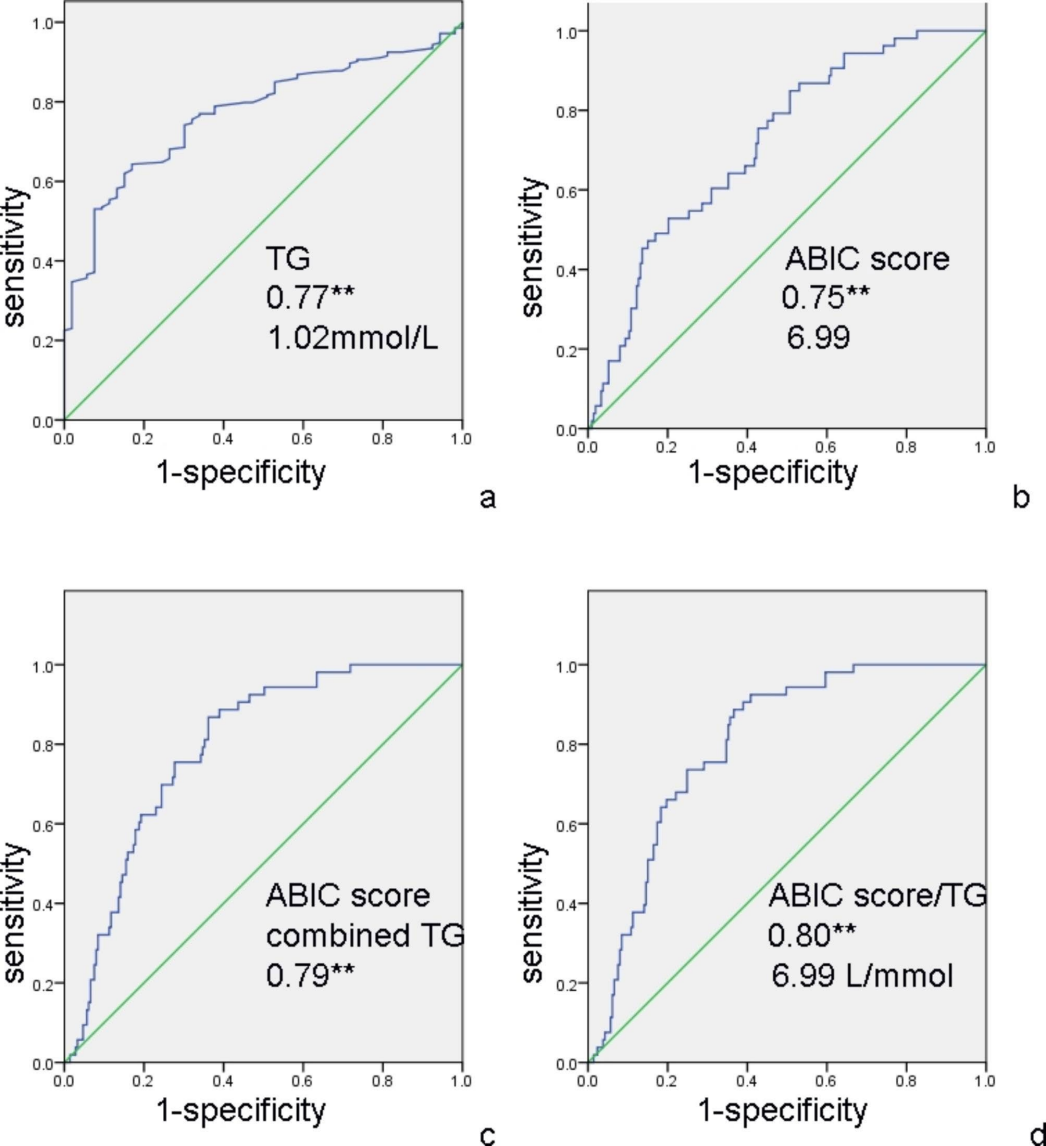
Receiver operating characteristic (ROC) curves of factors for the diagnosis of ALD-associated PLC. ALD: alcohol-associated liver disease; PLC: primary liver carcinoma; TG: triglyceride; ABIC: age-bilirubin-international normalized ratio (INR)-creatinine. AUC: area under the curve. (**a**) AUC and cutoff value of TG for the diagnosis of ALD-associated PLC. (**b**) AUC and cutoff value of the ABIC score for the diagnosis of ALD-associated PLC. (**c**) AUC of the combined ABIC score and TG for the diagnosis of ALD-associated PLC. (**d**) AUC and cutoff value of the ABIC score to TG ratio for the diagnosis of ALD-associated PLC. **P ≤ 0.01

To evaluate the reliability of the biomarkers related to ALD-associated PLC, the ABIC score was used as a positive predictive value, and TG was used as a negative predictive value because these parameters were more strongly related to the incidence of ALD-associated PLC than other factors. The AUC for TG was 0.77 (*P* < 0.01), with a TG level cutoff of 1.02 mmol/L based on the highest Youden index (0.47). The AUC for the ABIC score was 0.75 (*P* < 0.01) with an ABIC cutoff of 6.99 based on the highest Youden index (0.34). The combination of the ABIC score and TG yielded an AUC of 0.79 (*P* < 0.01). To obtain a cutoff point, the ABIC/TG ratio in ALD participants was analysed. The AUC for the ABIC/TG ratio was higher (AUC = 0.80, *P* < 0.01), with an optimal ABIC/TG cutoff of 6.99. The AUC for drinking time was 0.64 (*P* < 0.01), and the drinking time cutoff of 33.50 years was based on the highest Youden index (0.19). The AUC for average daily intake was 0.68 (*P* < 0.01), with an optimal daily drinking cutoff of 175.00 g/d. The above data are shown in Table 4; Fig. 4.

**Table 4:** Receiver operating characteristic (ROC) curve analysis of factors for the diagnosis of ALD-associated PLC

variables	AUC	sensitivity	1-specificity	youden index	cutoff value	*P*
drinking time (years)	0.64	0.51	0.32	0.19	33.50	< 0.01
average daily drinking (g/d)	0.68	0.45	0.15	0.30	175.00	< 0.01
Cho (mmol/L)	0.66	0.62	0.28	0.33	3.42	< 0.01
TG (mmol/L)	0.77	0.64	0.17	0.47	1.02	< 0.01
ABIC score	0.75	0.85	0.51	0.34	6.99	< 0.01
ABIC score combined TG	0.79	0.87	0.36	0.51	-	< 0.01
ABIC score/TG (L/mmol)	0.80	0.89	0.37	0.52	6.99	< 0.01

TG: triglyceride; Cho: cholesterol; ABIC: age-bilirubin-international normalized ratio (INR)-creatinine. AUC: area under the curve

## Discussion

Chronic alcohol intake changes the structure and function of the liver by triggering liver steatosis, hepatitis, cirrhosis and carcinogenesis [16]. The physiopathological mechanisms that are specific to ALD-associated PLC are as follows. (1) Acetaldehyde and its deleterious effect on proteins and DNA result in the reversion of normal liver cells into stem cells that can progress to cellular carcinoma [17, 18]. (2) The activity of reactive oxygen species (ROS) and cytochrome P450 2E1 (CYP2E1) are increased by alcohol stimulation, which induces damage to proteins and DNA and participates in carcinoma progression [19, 20]. (3) The induction of chronic inflammation and immunological system damage resulting from chronic drinking is related to carcinoma [21]. (4) Alcohol directly induces aberrant DNA methylation by inhibiting the synthesis of the methyl group donor S-adenosyl-L-methionine (SAMe); ultimately, aberrant methylation of DNA could contribute to ALD-associated PLC [22, 23].

In clinical research, age is a high risk factor for many malignancies, including ALD-associated PLC [21]. This study showed that participants who were 60 years or older had an increased risk of ALD-associated PLC. Previous studies have shown that chronic excessive drinking is a positive risk factor for liver carcinoma [24, 25]. The results showed that drinking time and average daily intake were related to PLC. The AUCs of drinking time and average daily intake for predicting PLC were 0.64 and 0.68, respectively.

Alcohol abuse results in lipid accumulation, including triglycerides and cholesterol, in hepatocytes. The single nucleotide rs738409 (G) allele, which is related to fatty liver disease, has been demonstrated to be a major genetic driver of liver carcinoma in ALD [17]. Lipid accumulation in hepatocytes is related to the microRNA miR-129-5p, which has been revealed in HCC [26, 27]. Genetic mutation promotes intracellular triglyceride accumulation and lipotoxicity in hepatocytes, which is associated with PLC. In addition, recent studies have shown that serum lipids are associated with some malignant diseases, including fatty liver-associated liver cancer, melanoma, prostate cancer, breast cancer, and gastrointestinal cancer [28–31]. In this study, serum lipid levels, including TG and Cho levels, were reduced in the ALD-associated-PLC group compared with the ALD-without-PLC group. The mechanism might be related to the malnutrition and genetic mutation induced by chronic heavy drinking.

The ABIC score is a tool to assess the severity and mortality of alcoholic hepatitis (AH) patients. Recently, the ABIC score was used in other diseases. The ABIC scoring system was estimated to be more effective than the MELD scoring system in the prognosis of the mortality of HBV-related acute-on-chronic liver failure [32]. Research indicated that percutaneous coronary intervention participants had a high risk of cardiac death with an ABIC score ≥ 7.985 [33]. Previous studies have shown that age and biomarkers of bilirubin, INR and creatinine were related to PLC incidence and progression, and it is reasonable that the ABIC scoring system might be a predictive index for ALD-associated PLC patients [34–38]. In this research, the ABIC score of PLC participants was higher than that of ALD-without-PLC participants (AUC = 0.75, *P* < 0.01), and ALD participants had a higher risk of PLC with an ABIC score ≥ 6.99. The ABIC/TG ratio of liver carcinoma patients was significantly higher than that of ALD-without-PLC participants (AUC = 0.80, *P* < 0.01). ALD participants had a higher risk of PLC with an ABIC/TG ratio ≥ 6.99.

The accumulation of lipids in hepatocytes has been demonstrated to be related to the progression of ALD [39]. The role of serum lipid levels in ALD is still controversial [40]. Furthermore, little research has been designed to detect the association between serum lipid levels in ALD and ALD-associated PLC. The ABIC/TG ratio was confirmed to have predictive value in ALD-associated PLC incidence.

### Comparisons with other studies and what does the current work add to the existing knowledge

Compared with previous studies, first, this is the first work in China to study the correlation between serum lipid levels and the incidence of ALD-associated PLC. To our knowledge, the results showed that TG and Cho were reduced in PLC participants with ALD. Second, the ABIC score was first used for predicting PLC in ALD, and the results showed that ABIC might have prognostic value in ALD. Third, the ABIC/TG ratio model was demonstrated to be effective in predicting the incidence of PLC, which has not been reported in previous studies.

### Study strengths and limitations

The cutoff value of the ABIC/TG ratio could be helpful for monitoring the status of ALD. The results showed reasonable performance for the prediction of incident PLC events. The feasible index could be used to predict ALD-associated PLC in clinical applications.

This research still has certain limitations that should be considered. First, it was conducted in a single center, and the sample was not large enough to symbolize the general situation. Second, some factors were not taken into account, such as plateletcrit (PCT), prothrombin index (PI), apolipoprotein A1, and alpha fetoprotein (AFP), which may be effective predictors of severity in ALD [41–43]. Third, some PLC participants’ biopsy results were incomplete, and histological characteristics could not be analyzed in the study. Fourth, we excluded participants with viral hepatitis, which creates bias when compared with other studies associated with ALD. In addition, outpatient information with ALD should be collected in future studies.

## Conclusion

Prognosis assessment is an essential step in evaluating lifestyle modifications and treatment needs to manage the risk of carcinogenesis. First, the study found that drinking time, average daily intake, TG and ABIC score are of diagnostic value for ALD-associated PLC. Second, the combination of the ABIC score and TG level was superior to a single factor in predicting ALD-associated PLC. The results showed that the ABIC/TG ratio was applicable to the prediction of carcinoma in ALD, and the AUC was higher than that of the combined factors. In clinical practice, the ABIC/TG ratio could be applied to better predict ALD-associated PLC. Health education is essential in ALD treatment, including quitting drinking as early as possible and nutritional support, which is necessary to maintain serum lipid levels in the normal range. In addition, a regular check-up including hepatic function, renal function, coagulation function and serum lipid levels seems to be necessary for ALD patients.

### Electronic supplementary material

Below is the link to the electronic supplementary material.

Supplementary Material 1

Supplementary Material 2

Supplementary Material 3

## Data Availability

The data of the current study are available from the corresponding author on reasonable request.

## Abbreviations

ALD: Alcohol-associated liver disease
HCC: Hepatocellular carcinoma
PLC: Primary liver carcinoma
TG: Triglyceride
Cho: Cholesterol
HDL-C: High-density lipoprotein cholesterol
LDL-C: Low-density lipoprotein cholesterol
ALT: Alanine aminotransferase
AST: Aspartate transaminase
GGT: Gamma-glutamyl transferase
AKP: Alkaline phosphatase
TBIL: Total bilirubin
DBIL: Direct bilirubin
PT-INR: International normalized ratio of prothrombin time
DF: Maddrey discriminant function
MELD: Model for End-stage Liver Disease
ABIC: Age-bilirubin-international normalized ratio (INR)-creatinine (ABIC)
GAHS: Glasgow alcoholic hepatitis scores
AUC: Area under the curve
