# Supplementary figures and images for: Increased age, bilirubin, international normalized ratio, and creatinine score to triglyceride ratio are associated with alcohol-associated primary liver carcinoma: a single-centered retrospective study
Source: Lipids Health Dis. 2023 Aug 4;22:117. doi: 10.1186/s12944-023-01888-y (PMC10401853; doi:10.1186/s12944-023-01888-y)

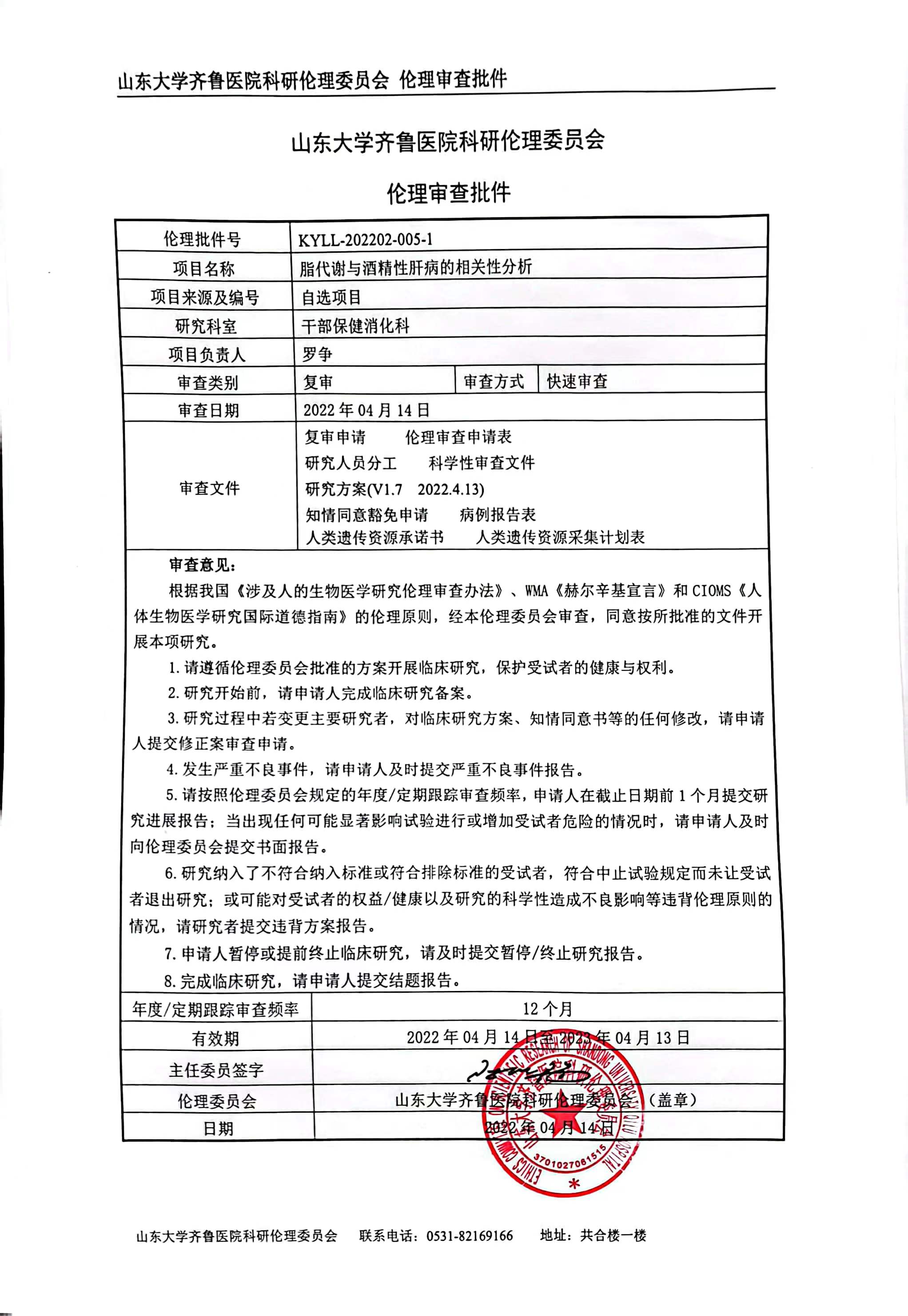

Supplement: Supplementary file 1 — Supplementary Material 1 [file 12944_2023_1888_MOESM1_ESM.jpg]

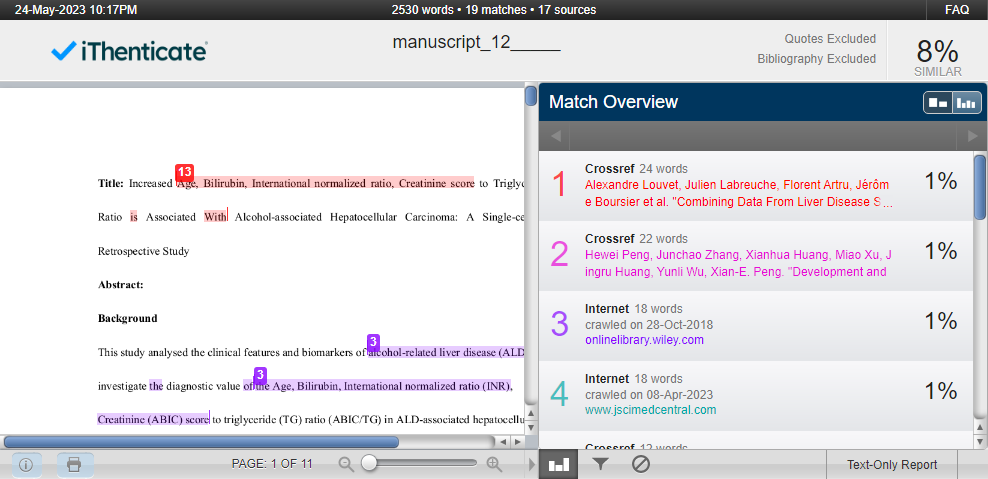

Supplement: Supplementary file 3 — Supplementary Material 3 [file 12944_2023_1888_MOESM3_ESM.png]
