# Supplementary material for: Increased age, bilirubin, international normalized ratio, and creatinine score to triglyceride ratio are associated with alcohol-associated primary liver carcinoma: a single-centered retrospective study
Source: Lipids Health Dis. 2023 Aug 4;22:117. doi: 10.1186/s12944-023-01888-y (PMC10401853; doi:10.1186/s12944-023-01888-y)
Supplement: Supplementary file 2 — Supplementary Material 2 [file 12944_2023_1888_MOESM2_ESM.pdf]

SPRINGER NATURE

# Author Services

# Editing Certificate

This document certifies that the manuscript

## Increased age-bilirubin-international normalized ratio-creatinine score to triglyceride ratio is associated with hepatocellular carcinoma in patients with alcohol-associated liver disease: a single-centered retrospective study

prepared by the authors

Zheng Luo

was edited for proper English language, grammar, punctuation, spelling, and overall style by one or more of the highly qualified native English speaking editors at SNAS.

This certificate was issued on **May 14, 2023** and may be verified on the SNAS website using the verification code **63DC-6491-3C76-2C3F-3F4F**.

Neither the research content nor the authors' intentions were altered in any way during the editing process. Documents receiving this certification should be English-ready for publication; however, the author has the ability to accept or reject our suggestions and changes. To verify the final SNAS edited version, please visit our verification page at secure.authorservices.springernature.com/certificate/verify. If you have any questions or concerns about this edited document, please contact SNAS at support@as.springernature.com.

SNAS provides a range of editing, translation, and manuscript services for researchers and publishers around the world. For more information about our company, services, and partner discounts, please visit authorservices.springernature.com.
